# Carbon Dots Fabrication: Ocular Imaging and Therapeutic Potential

**DOI:** 10.3389/fbioe.2020.573407

**Published:** 2020-09-25

**Authors:** Inyoung Garner, Riddhi Vichare, Ryan Paulson, Rajagopal Appavu, Siva K. Panguluri, Radouil Tzekov, Nurettin Sahiner, Ramesh Ayyala, Manas R. Biswal

**Affiliations:** ^1^MSPN Graduate Programs, Department of Pharmaceutical Sciences, Taneja College of Pharmacy, University of South Florida, Tampa, FL, United States; ^2^Department of Pharmaceutical Sciences, Taneja College of Pharmacy, University of South Florida, Tampa, FL, United States; ^3^Department of Ophthalmology, Morsani College of Medicine, University of South Florida, Tampa, FL, United States; ^4^Department of Chemistry, Canakkale Onsekiz Mart University, Canakkale, Turkey

**Keywords:** anti-vegf, ocular imaging, quantum dots, carbon dots, nanoparticle, fundus imaging, anti-bacterial

## Abstract

Vision loss is a major complication in common ocular infections and diseases such as bacterial keratitis, age-related macular degeneration (AMD) and diabetic retinopathy (DR). The prevalence of such ophthalmic diseases represents an urgent need to develop safe, effective, and long-term treatments. Current therapies are riddled with drawbacks and limitations which calls for the exploration of alternative drug delivery mechanisms. Toxicity of the inorganic metals and metal oxides used for drug delivery raise safety concerns that are alleviated with the alternate use of, a natural and organic polymer which is both biocompatible and environmentally friendly. Carbon dots (CDs) represent a great potential in novel biomedical applications due to their tunable fluorescence, biocompatibility, and ability to be conjugated with diverse therapeutic materials. There is a growing interest on the exploitation of these properties for drug delivery with enhanced bio-imaging. However, there are limited reports of CD applications for ophthalmic indications. In this review, we focus on the CD potential and the development of translational therapies for ophthalmic diseases. The current review presents better understanding of fabrication of CDs and how it may be useful in delivering anti-bacterial agents, anti-VEGF molecules as well as imaging for ophthalmic applications.

## Introduction

While a number of carbon allotropes are available such as graphene oxides, carbon nanotubes, and nanodiamonds, carbon dots (CDs) are the ideal choice for novel biomedical applications due to their advantages such as tunable fluorescence, excellent biocompatibility, and facile conjugation with diverse therapeutic materials (Ghosal and Ghosh, 2019; Sahiner et al., 2019). With their low-cost synthesis, CDs are an ideal solution to the search for alternatives in most biomedical applications. While the advantages of CDs in therapeutics and bioimaging have been thoroughly investigated, there are limited reports of their use in ophthalmic applications.

Common ocular infections such as bacterial keratitis or endophthalmitis require safe and effective antibiotic treatments. In the face of multi-drug antibiotic resistance, various nanomaterials such as silver, copper oxide, iron oxide, titanium oxide and zinc oxide particles have been garnering wide-spread attention for their novel anti-bacterial properties (Hajipour et al., 2012; Ananth et al., 2015; Beyth et al., 2015; Durán et al., 2016; Yadav et al., 2016; Muthukumar et al., 2017). However, the toxicity of such inorganic metals and metal oxides raise safety concerns that could be alleviated with CDs, a natural and organic polymer, both biocompatible and environmentally friendly (Vaz et al., 2017; Sahiner et al., 2019).

A common treatment for retinal vascular diseases such as exudative, or “wet” age-related macular degeneration (wAMD) and diabetic retinopathy (DR) is anti-VEGF therapy (Mandal et al., 2018). The greatest challenge for this therapy remains its administration to the posterior eye, presenting increased risks of infection, retinal detachment, and hemorrhage (Peng et al., 2017). CDs display an intrinsic anti-angiogenic profile which can be safely and effectively utilized to inhibit angiogenesis *in vitro* (Murugesan et al., 2007; Shereema et al., 2015). Often, such therapies can be tracked using fluorescent angiography (FA), a visualization of the retinal vasculature using a fluorescein dye. The disadvantages of traditional dyes such as a narrow excitation spectrum, photo-belching, and relatively high cost (Jensen, 2012; Qu et al., 2017) allow the CDs to shine as their tuned fluorescence can provide an excellent alternative.

While a variety of top-down and bottom-up methods are available for the synthesis of CDs, the feasibility and reproducibility of a selected method is the foremost of considerations. In addition to the selection of the method, there are many parameters that must be reviewed for the CD synthesis to yield a substantial therapeutic advantage. This work provides an overview of how CDs may be useful in delivering therapeutics such as anti-bacterial agents, anti-VEGF molecules, as well as bioimaging agents for ophthalmic indications.

## Antibacterial CDs

Biocompatible, antibacterial CDs can be fabricated from a wide range of materials such as Lawsonia inermis (the Henna plant), bovine serum albumin (BSA) and spermidine (Jian et al., 2017; Maruthapandi et al., 2019; Shahshahanipour et al., 2019). Furthermore, the use of precursors from biological origins such as amino acid, citric acid and so on in the synthesis of CDs can impart natural biocompatibility and antibacterial properties (Demirci et al., 2020). Modifications of CDs such as the surface functionalization of polyethyleneimine (PEI), colloidal polydopamine (PDA) and genipin have all led to an enhancement of their inhibitory effect on various strains of bacteria (Maruthapandi et al., 2019; Sahiner et al., 2019; Chu et al., 2020). Recent publications not only show that the potential of antibacterial CDs is being currently realized, but with a greater therapeutic advantage than their precursors and competitors.

### Antibacterial CDs for Ocular Infections

The bacterial strain, *Staphylococcus aureus* (*S. aureus*), is a common pathogen of eye infections like bacterial conjunctivitis, bacterial keratitis, and endophthalmitis (O’Callaghan, 2018). The most common treatment for such ocular infections is a broad-spectrum antibiotic, which can be ineffective against multi-drug resistance strains of *S. aureus* (MRSA) (Shanmuganathan et al., 2005; Lichtinger et al., 2012; Ong et al., 2013; Deguchi et al., 2018). Nitrogen (N)-doped CDs have shown antibacterial actions against both *S. aureus* and MRSA and are comparable in effectiveness to vancomycin on the MRSA-infected wounds in rats (Zhao et al., 2019) without impacting structural organization. The antibacterial mechanism proposed is the result of increased interactions between the positively charged CDs and the negatively charged surface of the bacterial membrane. While the concentration of N-doped CDs applied to the wound was higher than that of the vancomycin, N-doped CDs are produced from inexpensive raw materials, making them more cost-effective even at a higher concentration.

Topical treatment of super-cationic CDs from spermidine (SPDS-CD) has proved to be an effective inhibitor of multiple bacterial strains (including MRSA), exhibiting a lower minimal inhibitory concentration (MIC) than silver nanoparticles (AgNP) or PEI CDs (standards for strong antibacterial potency) (Jian et al., 2017). Although traditional eye drops are generally limited by poor corneal penetration and low bioavailability, this was not the case with SPDS-CDs. As a result of its super cationic property, SPDS-CDs induced an opening of the tight junctions in the corneal epithelium, increasing its permeability and therefore enhancing the bioavailability throughout the cornea. In fact, the performance of SPDS-CDs was comparable to a commercial eye drop formulation, sulfamethoxazole (SMX) at 10 times the marketed concentration. Comparing the efficacy of SPDS-CDs to other common antibiotics such as ciprofloxacin and vancomycin may further validate the therapeutic potential of SPDS-CDs.

### Synthesis Methods for CDs

Carbon dot synthesis can be categorized into (1) top-down or (2) bottom-up methods. The most recent publications display a trend of employing a bottom-up method for antibacterial CD synthesis such as hydrothermal synthesis, microwave synthesis, and thermal decomposition (Jian et al., 2017; Ghosal and Ghosh, 2019; Maruthapandi et al., 2019; Shahshahanipour et al., 2019; Zhao et al., 2019; Chu et al., 2020). Hydrothermal synthesis of CDs is a common method that is both eco-friendly and inexpensive (Ghosal and Ghosh, 2019). It involves a heating of the reaction mixture at a temperature of 150–250°C for 40 min to 12 h (Jian et al., 2017; Ghosal and Ghosh, 2019; Maruthapandi et al., 2019; Shahshahanipour et al., 2019; Zhao et al., 2019; Chu et al., 2020). In contrast, a microwave synthesis frequently takes under 5 min using microwaves at 500–800 W to break down the carbon bonds of the organic source (Ghosal and Ghosh, 2019). This method is especially useful for quickly and efficiently delivering CDs that are uniform in size (Ghosal and Ghosh, 2019; Sahiner et al., 2019). On the other hand, thermal decomposition method is the best known synthesis for CDs, as it offers a simple, cost-effective, and large-scale production with short reaction times (Ghosal and Ghosh, 2019). Regardless of the specific indications for which a CD is being synthesized, the basic principles of each method remain the same while different temperatures, durations, and power are harnessed.

### Key Parameters for Antibacterial CD Synthesis

To synthesize antibacterial CDs with a substantial therapeutic advantage that can compete with the current market, one should focus on the size, surface charge, and functionalization of the CD. An important mechanism of nanoparticle (NP)-mediated microbial resistance is the disruption of biofilm formation (Wang et al., 2017). This is facilitated by the small size and higher surface area-to-mass ratio of the NP (Wang et al., 2017). In addition, CDs with neutral or positive charges have a greater antibacterial efficacy than the negatively charged CDs due to the facilitated interactions with the negatively charged lipid membrane of bacterial cells (Verma et al., 2019). The generation of reactive oxygen species (ROS), as a bactericidal mechanism, is greater in uncharged versus positively charged CDs due to the difference in the availability of negatively charged functional groups (Verma et al., 2019). For ophthalmic indications, CDs with a positive zeta potential has been shown to better cross the corneal epithelium, resulting in a high bioavailability, something atypical in traditional topical treatments (Jian et al., 2017). Surface functionalization is also important for enhancing the antibacterial properties of CDs and their distribution across the cornea. The conjugation of PEI to Arginine-CDs (A-CDs) results in blood compatible CDs with reduced minimum inhibitory concentration (MIC) and minimum bacterial concentration (MBC) of *S. aureus* by 2.5-fold and 10-fold, respectively (Sahiner et al., 2019). Together, the size, charge and functionalization of CDs significantly affects the antibacterial potency of CDs and therapeutic potential.

## CDs for Ocular Neovascularization

The abnormal growth of blood vessels in the eye may originate from cornea, retina and choroid. New blood vessels originating from the choroid layer is a major implication in wAMD. New blood vessels originating from retina are the clinical phenotype for DR and retinopathy of prematurity (ROP). In either case, local secretion of VEGF induces uncontrolled neovascularization that ultimately causes irreversible damage to the retina causing a loss of vision that cannot be restored. The ability to functionalize the versatile surface of CDs with anti-VEGF agents presents unique opportunities to investigate their effect in inhibiting ocular neovascularization. Anti-VEGF agents have long been tested in preclinical models of wAMD (Biswal et al., 2018), DR and ROP (Biswal et al., 2014) and in a topical CD formulation, may provide less invasive and more cost-effective treatments for ocular neovascular diseases.

### CDs for Anti-VEGF Therapy

Recently, the delivery of anti-VEGF agents through topical formulations as an alternative to the more invasive intravitreal injections has been garnering attention (Dastjerdi et al., 2009; Wilkinson-Berka and Deliyanti, 2016; De Cogan et al., 2017). The success of Vasotide, a small cyclic retro-inverted peptidomimetic in an eye drop form shows the potential of such applications. Vasotide successfully decreased angiogenesis in two mice models of retinopathy and a monkey model of human wet-AMD (Sidman et al., 2015). Traditionally, topical formulations penetrate the corneal epithelium to diffuse throughout the anterior segment with a limited capacity, and does not achieve therapeutic concentrations in the posterior eye (Mandal et al., 2018). However, small molecules, such as Vasotide, could be absorbed through the conjunctiva, and have the potential to reach the vitreous and the retina (Sidman et al., 2015; Rodrigues et al., 2018). The results of this study demonstrate that the efficacy of a topical formulation is not limited to the anterior segment of the eye.

More recently, CDs were successfully functionalized with anti-VEGF aptamers to effectively reduce angiogenesis in an *in vitro* model of CNV. The authors found the anti-angiogenic effect of the anti-VEGF-CDs to be similar to that of two anti-VEGF agents available on the current market, bevacizumab and aflibercept (Shoval et al., 2019). As expected, the anti-VEGF-CDs did not exhibit any *in vitro* cytotoxicity to human fibroblasts, ARPE-19 cells, and human embryonic stem cell-derived photoreceptor cells, or any *in vivo* cytotoxicity to Long-Evans pigmented rats (Shoval et al., 2019). Both Sidman et al. (2015) and Shoval et al. (2019) have demonstrated a remarkable ability to effectively deliver anti-VEGF agents to the posterior eye using non-invasive topical treatments. This represents a breakthrough in terms of a treatment that will improve a patient’s quality of life, especially those in the aging population that continue to suffer from debilitating diseases such as wAMD and DR.

### Key Parameters for Anti-VEGF CD Synthesis

The size of CDs for ocular neovascularization is of great importance, especially for a topical formulation. As previously mentioned, the size of the molecules intended for delivery is critical for passage through the conjunctiva route. Vasotide, for example, had a molecular weight of 26 kDa, for reference, a peptide of 20 kDa (when folded into a single domain protein) is roughly equal to the size of 1.78 nm (Erickson, 2009). Because an anti-VEGF functionalized CD of this size is unlikely, further investigations are needed to find the ideal CD size for anterior administration and posterior indications. Studies indicate molecules of molecular weight as large as 66 kDa (∼7.2 nm) can diffuse through the human cornea while those larger than 76 kDA are severely limited in their diffusion to the retina (Charalel et al., 2012; Kim et al., 2014). For the synthesis of anti-VEGF-CDs, particles should generally be small enough to pass through the specific ocular barrier while also being large enough to avoid accelerated clearance from its destination, the posterior eye.

In addition to size, the hydrophobicity and fluorescence quenching should also be taken into account. Shoval et al. (2019) found the anti-VEGF functionalization resulted in hydrophilic regions on their hydrophobic CDs creating an amphiphilic complex critical for delivery. The complex could easily penetrate the hydrophobic corneal epithelium and the hydrophilic corneal stroma. Although the conjugation of anti-VEGF aptamers is important, their effect on CD luminescence is a concern. Ideally, surface functionalization should be achieved in a way that preserves the photoluminescence properties of the CDs for bioimaging. Basiri et al. found the conjugation of VEGF resulted in varying degrees of fluorescence quenching depending on the concentration of the aptamer (Basiri et al., 2020). A similar effect has been seen in conjugation of proteins to semiconductor and carbon quantum dots (Devi and Tyagi, 2019) and demonstrates the importance of considering such factors during the functionalization of CDs.

## CDs for Ocular Bioimaging

**FIGURE 1:**
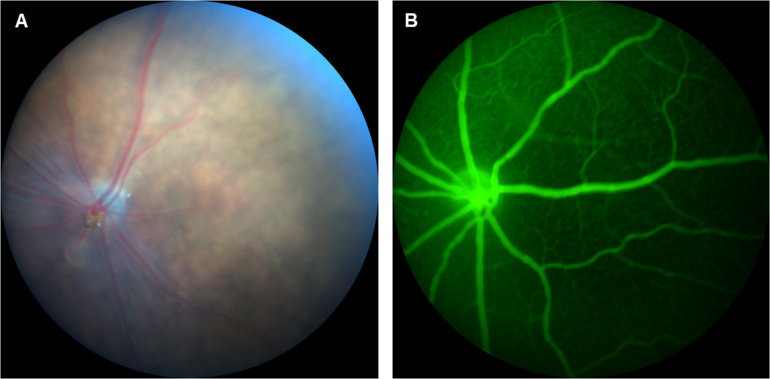
**(A)** Fundus imaging and **(B)** fluorescein angiography (FA) of mouse retina.

As the number of reports continue to increase on CDs for enhanced bioimaging applications, their translation to fluorescein angiography (FA) becomes more relevant. FA is a method of visualizing microvascular changes in the retina for both animal research and clinical applications (Figure 1). Such applications include the tracking of a therapeutic delivery or the diagnosis of ophthalmic diseases such as wAMD and DR (Kim et al., 2013; Qu et al., 2017). However, this technique is currently limited by the disadvantages of fluorescent dyes such as a narrow excitation spectrum, photo-bleaching, and relatively high cost (Jensen, 2012; Qu et al., 2017). Unlike organic dyes, CDs are known for their high photostability and intrinsic fluorescence which is tunable rendering CDs an excellent alternative to fluorescent dyes in FFA applications.

### CDs for Fluorescein Angiography

Qu et al. (2017) demonstrated the use of heteroatom-doped CDs in FA with advantages such as a longer wavelength excitation and emission. A simple, 8-h hydrothermal synthesis yielded in selenium and nitrogen co-doped CDs with a high green fluorescence and low cytotoxicity. Post-administration to C57BL/6J mice, the co-doped CDs produced a clear FFA image of the retinal vasculature as well as details of the capillary bed, providing the proof of concept for CDs as high-performance fluorescent imaging agents for angiography. However, a limitation of this study remains the extent of cytotoxicity characterization, as only *in vitro* results in murine liver cells were reported. No further data was available on the structural organization of ocular tissues post *in vivo* studies. In addition, this study may be improved with a pharmacokinetic characterization of the CDs, including bioavailability, retention time as well as the clearance from the posterior eye.

Another study demonstrated the synthesis of red-emissive nitrogen-doped CDs for enhanced bioimaging (Karakoçak et al., 2018). Their synthesis protocol was the result of an optimization of three different factors, including the ratio of amine to acid precursor molar ratio, duration of microwave analysis, and the concentration of citric acid. The optimization resulted in CDs with a red fluorescence of 600–700 nm. *In vitro* cytotoxicity results revealed that LD_50_ of synthesized CDs to both retinal and lens epithelial cells was approximately 0.6 mg/mL (10 times the concentration needed for *in vitro* imaging). The *ex vivo* results of porcine eyes demonstrated the effective diffusion and distribution of the intravitreally injected CDs throughout multiple layers of the eye. As previously mentioned, this study may also be improved by the evaluation of CD pharmacokinetics, as the percentage and duration of bioavailability as well as the route of clearance which are important factors to consider in the development of bioimaging agents.

### CD Synthesis Parameters for FA

The primary parameters of CD synthesis for FA remain the excitation and emission of the fluorescence which determine the viability of CD applications. Although CDs possess a native blue fluorescence, the low penetration depth along with biological tissue interference (autofluorescence) limits their use in bioimaging applications (Qu et al., 2017; Karakoçak et al., 2018). In addition, the excitation of CD’s native fluorescence occurs in the UV spectra, leading to severe photodamage. Therefore, modifications must be made to fabricate CDs with excitations and emissions of longer wavelengths.

As previously mentioned, Qu et al. (2017) synthesized selenium and nitrogen co-doped CDs with low cytotoxicity and a high fluorescence. Interestingly, the addition of Se to the N-doped CDs expanded the absorption from the UV to the visible light spectrum (∼497 nm), resulting in a strong green fluorescence with a deep penetration. Another study optimized three different parameters, including the ratio of amine to acid precursor molar ratio, duration of microwave pyrolysis, and the concentration of citric acid to produce CDs with a deep red fluorescence (Karakoçak et al., 2018). The resulting emission type was ideal for optimal bioimaging due the lack of tissue interference. Authors found that the emission around 600–700 nm increased with the increasing amine to acid ratio, and while the reaction time had a positive effect at a high amine to acid ratio, it did not significantly affect the resulting emission (Karakoçak et al., 2018). Karakocak et al. also found the neutral and positive charged CDs exhibited a stronger fluorescence, and more likely to be excited at longer wavelengths.

## Perspective

This work provides an overview of the major CD applications in nanobiomedicine and their translatability to ophthalmic indications which is summarized in Table 1. CDs have the potential to become inexpensive, biocompatible and versatile treatments that can be easily administered or received. Researchers have already demonstrated the ability to topically deliver antibacterial or anti-VEGF CDs to treat ocular infections or angiogenesis. In addition, the immense progress made in CDs for bioimaging translates to their application in FA. The intrinsic fluorescence of CDs facilitates the concept of theranostics in which diagnostics and therapy are offered simultaneously. More importantly, this review highlights the various properties of CDs and the modifications critical for honing those properties in a variety of biomedical applications. Although there is a limited number of publications available on the use of CDs in the eye, the methods and synthesis of CDs in other fields are certainly translatable to the ocular field. By providing information regarding the critical parameters for optimizing CD synthesis for specific indications, we invite others to use the available information and gather what is necessary to realize even further potential of applications of CDs in ophthalmic indications.

**TABLE 1:** Summary of strengths and weaknesses of carbon dot applications.

**CD Application**	**Strengths**	**Weaknesses**
Anti-bacterial	• Biocompatibility	• May require higher concentrations for substantial therapeutic effect.
	• Low cost	
	• Higher efficacy	
	• Effective against MDR-bacteria.	
Anti-VEGF	• Biocompatibility	• Penetration capacity may differ based on formulation.
	• Low cost	
	• Non-invasive when applied topically	• Anti-VEGF functionalization may decrease fluorescence.
	• Provides therapy tracking through fluorescence	
Fundus Angiography	• Biocompatibility	• Native fluorescence occurs in the UV spectra.
	Low cost	
	• Tunable fluorescence	• Functionalization may decrease fluorescence depending on the moiety.
	• Photostability	
	• Increased resolution	

## Author Contributions

IG and MB wrote the article. RV, RP, RA, SP, NS, RT, and RA provide concepts and helped editing the manuscript. All authors contributed to the article and approved the submitted version.

## Conflict of Interest

The authors declare that the research was conducted in the absence of any commercial or financial relationships that could be construed as a potential conflict of interest.

## References

[B1] Ananth A. Dharaneedharan S. Heo M.-S. Mok Y. S. (2015). Copper oxide nanomaterials: synthesis, characterization and structure-specific antibacterial performance. *Chem. Eng. J.* 262 179–188. 10.1016/j.cej.2014.09.083

[B2] Basiri H. Abouei Mehrizi A. Ghaee A. Farokhi M. Chekini M. Kumacheva E. (2020). Carbon dots conjugated with vascular endothelial growth factor for protein tracking in angiogenic therapy. *Langmuir* 36 2893–2900. 10.1021/acs.langmuir.9b03980 32125865

[B3] Beyth N. Houri-Haddad Y. Domb A. Khan W. Hazan R. (2015). Alternative antimicrobial approach: nano-antimicrobial materials. *Evid. Based Complement. Alternat. Med*. 2015:246012. 10.1155/2015/246012 25861355 PMC4378595

[B4] Biswal M. R. Prentice H. M. Dorey C. K. Blanks J. C. (2014). A hypoxia-responsive glial cell–specific gene therapy vector for targeting retinal neovascularization. *Invest. Ophthalmol. Vis. Sci.* 55 8044–8053. 10.1167/iovs.14-13932 25377223 PMC4263136

[B5] Biswal M. R. Prentice H. M. Smith G. W. Zhu P. Tong Y. Dorey C. K. (2018). Cell-specific gene therapy driven by an optimized hypoxia-regulated vector reduces choroidal neovascularization. *J. Mol. Med.* 96 1107–1118. 10.1007/s00109-018-1683-0 30105447

[B6] Charalel R. A. Engberg K. Noolandi J. Cochran J. R. Frank C. Ta C. N. (2012). Diffusion of protein through the human cornea. *Ophthalmic. Res.* 48 50–55. 10.1159/000329794 22398578 PMC3569487

[B7] Chu X. Wu F. Sun B. Zhang M. Song S. Zhang P. (2020). Genipin cross-linked carbon dots for antimicrobial, bioimaging and bacterial discrimination. *Colloids. Surf. B Biointerfaces* 190:110930. 10.1016/j.colsurfb.2020.110930 32146275

[B8] Dastjerdi M. H. Al-Arfaj K. M. Nallasamy N. Hamrah P. Jurkunas U. V. Pineda R. (2009). Topical bevacizumab in the treatment of corneal neovascularization: results of a prospective, open-label, non-comparative study. *Arch. Ophthalmol.* 127 381–389. 10.1001/archophthalmol.2009.18 19365012 PMC2703579

[B9] De Cogan F. Hill L. J. Lynch A. Morgan-Warren P. J. Lechner J. Berwick M. R. (2017). Topical delivery of anti-vegf drugs to the ocular posterior segment using cell-penetrating peptides. *Invest. Ophthalmol. Vis. Sci*. 58 2578–2590. 10.1167/iovs.16-20072 28494491

[B10] Deguchi H. Kitazawa K. Kayukawa K. Kondoh E. Fukumoto A. Yamasaki T. (2018). The trend of resistance to antibiotics for ocular infection of *Staphylococcus aureus*, coagulase-negative staphylococci, and *Corynebacterium* compared with 10-years previous: a retrospective observational study. *PLoS One* 7:13. 10.1371/journal.pone.0203705 30192856 PMC6128643

[B11] Demirci S. McNally A. B. Ayyala R. S. Lawson L. B. Sahiner N. (2020). Synthesis and characterization of nitrogen-doped carbon dots as fluorescent nanoprobes with antimicrobial properties and skin permeability. *J. Drug Deliv. Sci. Technol.* 59:101889 10.1016/j.jddst.2020.101889

[B12] Devi S. Tyagi S. (2019). Fluorescent determination of trinitrotoluene with bovine serum albumin mediated enhancement of thioglycolic acid capped cadmium selenium quantum dots. *Instrum. Sci. Technol.* 47 292–311. 10.1080/10739149.2018.1531019

[B13] Durán N. Durán M. de Jesus M. B. Seabra A. B. Fávaro W. J. Nakazato G. (2016). Silver nanoparticles: a new view on mechanistic aspects on antimicrobial activity. *Nanomedicine* 12 789–799. 10.1016/j.nano.2015.11.016 26724539

[B14] Erickson H. P. (2009). Size and shape of protein molecules at the nanometer level determined by sedimentation, gel filtration, and electron microscopy. *Biol. Proced. Online* 11 32–51. 10.1007/s12575-009-9008-x 19495910 PMC3055910

[B15] Ghosal K. Ghosh A. (2019). Carbon dots: the next generation platform for biomedical applications. *Mater. Sci. Eng. C Mater. Biol. Appl.* 96 887–903. 10.1016/j.msec.2018.11.060 30606603

[B16] Hajipour M. J. Fromm K. M. Ashkarran A. A. De Aberasturi D. J. de Larramendi I. R. Rojo T. (2012). Antibacterial properties of nanoparticles. *Trends Biotechnol.* 30 499–511. 10.1016/j.tibtech.2012.06.004 22884769

[B17] Jensen E. C. (2012). Use of fluorescent probes: their effect on cell biology and limitations. *Ana. t Rec. (Hoboken)* 295 2031–2036. 10.1002/ar.22602 23060362

[B18] Jian H.-J. Wu R.-S. Lin T.-Y. Li Y.-J. Lin H.-J. Harroun S. G. (2017). Super-cationic carbon quantum dots synthesized from spermidine as an eye drop formulation for topical treatment of bacterial keratitis. *ACS Nano* 11 6703–6716. 10.1021/acsnano.7b01023 28677399

[B19] Karakoçak B. B. Liang J. Kavadiya S. Berezin M. Y. Biswas P. Ravi N. (2018). Optimizing the synthesis of red-emissive nitrogen-doped carbon dots for use in bioimaging. *ACS Appl. Nano Mater*. 1 3682–3692. 10.1021/acsanm.8b00799

[B20] Kim D. Y. Fingler J. Zawadzki R. J. Park S. S. Morse L. S. Schwartz D. M. (2013). Optical imaging of the chorioretinal vasculature in the living human eye. *Proc. Natl. Acad. Sci. U.S.A.* 110 14354–14359. 10.1073/pnas.1307315110 23918361 PMC3761584

[B21] Kim Y.-C. Chiang B. Wu X. Prausnitz M. R. (2014). Ocular delivery of macromolecules. *J. Control. Release* 190 172–181. 24998941 10.1016/j.jconrel.2014.06.043 PMC4142116

[B22] Lichtinger A. Yeung S. N. Kim P. Amiran M. D. Iovieno A. Elbaz U. (2012). Shifting trends in bacterial keratitis in toronto: an 11-year review. *Ophthalmology* 119 1785–1790. 10.1016/j.ophtha.2012.03.031 22627118

[B23] Mandal A. Pal D. Agrahari V. Trinh H. M. Joseph M. Mitra A. K. (2018). Ocular delivery of proteins and peptides: challenges and novel formulation approaches. *Adv. Drug Deliv. Rev*. 126 67–95. 10.1016/j.addr.2018.01.008 29339145 PMC5995646

[B24] Maruthapandi M. Natan M. Jacobi G. Banin E. Luong J. H. T. Gedanken A. (2019). Antibacterial activity against methicillin-resistant *Staphylococcus aureus* of colloidal polydopamine prepared by carbon dot stimulated polymerization of dopamine. *Nanomaterials (Basel)* 9:1731 10.3390/nano9121731 31817151 PMC6955702

[B25] Murugesan S. Mousa S. A. O’Connor L. J. Lincoln D. W. Linhardt R. J. (2007). Carbon inhibits vascular endothelial growth factor- and fibroblast growth factor-promoted angiogenesis. *FEBS Lett.* 581 1157–1160. 10.1016/j.febslet.2007.02.022 17331505 PMC1994254

[B26] Muthukumar H. Chandrasekaran N. I. Naina Mohammed S. Pichiah S. Manickam M. (2017). Iron oxide nano-material: physicochemical traits and in vitro antibacterial propensity against multidrug resistant bacteria. *J. Ind. Eng. Chem.* 45 121–130. 10.1016/j.jiec.2016.09.014

[B27] O’Callaghan R. J. (2018). The pathogenesis of *Staphylococcus aureus* eye infections. *Pathogens* 7:9. 10.3390/pathogens7010009 29320451 PMC5874735

[B28] Ong S. J. Huang Y.-C. Tan H.-Y. Ma D. H. K. Lin H.-C. Yeh L.-K. (2013). *staphylococcus aureus* keratitis: a review of hospital cases. *PLoS One* 8:e80119. 10.1371/journal.pone.0080119 24244625 PMC3823797

[B29] Peng Y. Tang L. Zhou Y. (2017). Subretinal injection: a review on the novel route of therapeutic delivery for vitreoretinal diseases. *Ophthalmic. Res.* 58 217–226. 10.1159/000479157 28858866

[B30] Qu D. Miao X. Wang X. Nie C. Li Y. Luo L. (2017). Se & N co-doped carbon dots for high-performance fluorescence imaging agent of angiography. *J. Mater. Chem. B* 5 4988–4992. 10.1039/c7tb00875a 32264015

[B31] Rodrigues G. A. Lutz D. Shen J. Yuan X. Shen H. Cunningham J. (2018). Topical drug delivery to the posterior segment of the eye: a;ddressing the challenge of preclinical to clinical translation. *Pharm. Res*. 35:245. 10.1007/s11095-018-2519-x 30374744 PMC6208585

[B32] Sahiner N. Suner S. S. Sahiner M. Silan C. (2019). Nitrogen and sulfur doped carbon dots from amino acids for potential biomedical applications. *J. Fluoresc.* 29 1191–1200. 10.1007/s10895-019-02431-y 31502060

[B33] Shahshahanipour M. Rezaei B. Ensafi A. A. Etemadifar Z. (2019). An ancient plant for the synthesis of a novel carbon dot and its applications as an antibacterial agent and probe for sensing of an anti-cancer drug. *Mater. Sci. Eng. C* 98 826–833. 10.1016/j.msec.2019.01.041 30813088

[B34] Shanmuganathan V. A. Armstrong M. Buller A. Tullo A. B. (2005). External ocular infections due to methicillin-resistant *Staphylococcus aureus* (MRSA). *Eye (Lond.)* 19 284–291. 10.1038/sj.eye.6701465 15375372

[B35] Shereema R. M. Sruthi T. V. Kumar V. B. S. Rao T. P. Shankar S. S. (2015). Angiogenic profiling of synthesized carbon quantum dots. *Biochemistry* 54 6352–6356. 10.1021/acs.biochem.5b00781 26371545

[B36] Shoval A. Markus A. Zhou Z. Liu X. Cazelles R. Willner I. (2019). Anti-VEGF-aptamer modified c-dots-a hybrid nanocomposite for topical treatment of ocular vascular disorders. *Small* 15:e1902776. 10.1002/smll.201902776 31402576

[B37] Sidman R. L. Li J. Lawrence M. Hu W. Musso G. F. Giordano R. J. (2015). The peptidomimetic vasotide targets two retinal VEGF receptors and reduces pathological angiogenesis in murine and nonhuman primate models of retinal disease. *Sci. Transl. Med.* 7:309ra165. 10.1126/scitranslmed.aac4882 26468327 PMC4787616

[B38] Vaz R. Bettini J. Júnior J. G. F. Lima E. D. S. Botero W. G. Santos J. C. C. (2017). High luminescent carbon dots as an eco-friendly fluorescence sensor for Cr(VI) determination in water and soil samples. *J. Photochem. Photobiol. A Chem.* 346 502–511. 10.1016/j.jphotochem.2017.06.047

[B39] Verma A. Arshad F. Ahmad K. Goswami U. Samanta S. K. Sahoo A. K. (2019). Role of surface charge in enhancing antibacterial activity of fluorescent carbon dots. *Nanotechnology* 31:095101. 10.1088/1361-6528/ab55b8 31703210

[B40] Wang L. Hu C. Shao L. (2017). The antimicrobial activity of nanoparticles: present situation and prospects for the future. *Int. J. Nanomed.* 12 1227–1249. 10.2147/IJN.S121956 28243086 PMC5317269

[B41] Wilkinson-Berka J. L. Deliyanti D. (2016). The potential of anti-VEGF (vasotide) by eye drops to treat proliferative retinopathies. *Ann. Transl. Med.* 4 S41–S41. 10.21037/atm.2016.10.27 27868009 PMC5104608

[B42] Yadav H. M. Kim J.-S. Pawar S. H. (2016). Developments in photocatalytic antibacterial activity of nano TiO2: a review. *Korean J. Chem. Eng.* 33 1989–1998. 10.1007/s11814-016-0118-2

[B43] Zhao C. Wang X. Wu L. Wu W. Zheng Y. Lin L. (2019). Nitrogen-doped carbon quantum dots as an antimicrobial agent against *Staphylococcus* for the treatment of infected wounds. *Colloids Surf. B Biointerfaces* 179 17–27. 10.1016/j.colsurfb.2019.03.042 30928801

